# Synergistic inhibitory effects of capsaicin combined with cisplatin on human osteosarcoma in culture and in xenografts

**DOI:** 10.1186/s13046-018-0922-0

**Published:** 2018-10-16

**Authors:** Yang Wang, Xu Deng, Chang Yu, Guosheng Zhao, Jing Zhou, Ge Zhang, Ming Li, Dianming Jiang, Zhengxue Quan, Yuan Zhang

**Affiliations:** 1 Department of Orthopedic Surgery, The First Affiliated Hospital of Chongqing Medical University, Chongqing, 400016 People’s Republic of China; 2 Department of Orthopedic Surgery, Ministry of Education Key Laboratory of Child Development and Disorders, Key Laboratory of Pediatrics in Chongqing, China International Science and Technology Cooperation Base of Child Development and Critical Disorders, Children’s Hospital of Chongqing Medical University, Chongqing, 400014 People’s Republic of China; 3 Department of Orthopedic Surgery, The Third Affiliated Hospital of Chongqing Medical University, Chongqing, 401120 People’s Republic of China

**Keywords:** Combination therapy, Capsaicin, Cisplatin, Apoptosis, Autophagy

## Abstract

**Background:**

The combination of phytochemicals with chemotherapy drugs is an emerging new strategy for cancer therapy to increase antitumor responses.

**Methods:**

The present study investigates the effect of the combination of capsaicin (CAP) with cisplatin (DDP) and the potential underlying anticancer mechanisms in osteosarcoma (OS) cells in vitro and in vivo.

**Results:**

Cell viability assays and isobolographic analyses demonstrated that the combination of CAP and DDP showed synergistic cytotoxic effects on OS cells. We chose relatively low concentrations of CAP (100 μM) and DDP (16.7 μM) for subsequent experiments. Generally, the combination of CAP and DDP had significant effects on apoptosis induction, cell cycle arrest and cell invasion inhibition in OS cells compared with the individual-treatment groups and the control group. Moreover, cotreatment with CAP and DDP triggered prosurvival autophagy through reactive oxygen species (ROS)/JNK and p-AKT/mTOR signaling in OS cells. The combination regimen of CAP and DDP also inhibited tumor growth in an OS xenograft model.

**Conclusion:**

These results suggest that the combination of CAP and DDP has strong inhibitory effects on OS cells and identify CAP as a promising agent for supplementing standard chemotherapy and possible future targeted therapy in OS.

## Background

Osteosarcoma (OS) is the most common primary malignant tumor of the bone in children and adolescents [1]. Great progress has been made in the therapy of OS due to the utilization of neoadjuvant chemotherapy and radiotherapy in combination with surgical resection. Overall survival has increased to 60–75% and has remained the same for the last two decades [2]. Unfortunately, the prognosis of OS with metastasis is still poor; only 30% patients with metastatic OS achieve 5-year tumor-free survival [3].

Cisplatin (cis-diamminedichloroplatinum II, DDP) is a common and effective chemotherapeutic drug used in the treatment of various human solid tumors, including bladder cancer, cervical cancer, small cell lung cancer and gastric cancer [4]. DDP treatment is considered a useful chemotherapeutic method for preoperative induction therapy for OS with an improved survival rate [5]. The general mechanism by which DDP kills cancer cells has been elucidated. Briefly, DDP induces DNA intrastrand cross links between adjacent purines, which results in DNA damage that leads to the inhibition of tumor cell invasion and initiation of apoptosis, or programmed cell death [6]. DDP has an obvious killing effect on osteosarcoma cells; however, the toxicity and acquisition of intrinsic resistance by OS cells after long-term application of DDP remain major obstacles [7]. Recently, some novel compounds, such as platinum complexes and vanadium complexes, have been developed that exhibit efficacy against human OS cell lines and even some chemoresistant OS cell lines. These compounds may represent a new class of potent anti-OS agents but had limited efficacy under experimentally controlled conditions. Furthermore, the putative mechanisms and biosafety of these novel compounds still need to be elucidated in future research [8–10]. Therefore, there is an urgent need to develop a more effective and safe treatment strategy that combines a low dosage of DDP, one of the gold standard drugs in OS treatment, with other agents to decrease DDP-related side effects and chemoresistance.

Phytochemicals are a series of compounds that are extracted and purified from plants such as vegetables, fruits, spices, and grains. Many studies have demonstrated the pharmacological activities of phytochemicals, including antioxidant [11], antimicrobial [12], antidiabetic [13], and anti-inflammatory effects [14]. Most recently, the anticancer and chemoprevention properties of phytochemicals have attracted increasing interest from oncology researchers due to their low intrinsic toxicity in normal cells but prominent effects in cancerous cells [15]. Phytochemicals can exhibit diverse inhibitory effects on the initiation, promotion, progression, invasion and metastasis of cancer [16, 17]. Recent studies have shown that phytochemicals can restore the sensitivity of cancer cells to conventional chemotherapeutic drugs [18]. Synergistic or additional effects of combinations of DDP and phytochemical compounds in cancer cells with acceptable side effects have also been demonstrated [19, 20]. Capsaicin (trans-8-methyl-N-vanillyl-6-nonenamide, CAP) is one of the major pungent ingredients of red pepper and has been widely used in clinical medicine for the treatment of pain and inflammation caused by various diseases [21]. In addition, numerous studies and animal experiments have demonstrated the anticancer and chemopreventive properties of CAP [22]. Our previous study showed that CAP has profound in vitro and in vivo antiproliferative effects against human OS cells. However, apoptotic effects in OS cells were only observed upon treatment with a relatively high concentration of CAP [23]. Thus, we hypothesized that the combination of subtoxic concentrations of the phytochemical CAP and the conventional chemotherapeutic DDP could exhibit significant killing effects in OS cells. Here, we demonstrated that CAP synergistically potentiates the anticancer activity of DDP in OS cells in vitro and that the combination of CAP and DDP inhibits tumor growth more significantly than the control or any other single-administration group in the OS xenograft model. The possible molecular pathway underlying this effectiveness is also discussed. The present results suggest that CAP could serve as a candidate for further development as a chemotherapy adjuvant for the treatment of OS.

## Methods

### Reagents and chemicals

Capsaicin (trans-8-methyl-N-vanillyl-6-nonenamide) was purchased from Sigma-Aldrich (St. Louis, MO, USA). Cisplatin (DDP) was purchased from Qilu Pharm (Jinan, China). Fetal bovine serum (FBS) and Dulbecco’s-modified Eagle’s medium (DMEM) were purchased from HyClone (Logan, UT, USA). The cell viability and cytotoxicity test kit, namely Cell Counting Kit-8 (CCK-8), was purchased from Dojindo Molecular Technologies (Kimamoto, Japan). The Annexin V-FITC/propidium iodide (PI) double-staining test kit was purchased from KeyGen Biotech (Nanjing, China). RIPA lysis buffer, phenylmethanesulfonyl fluoride (PMSF), the bicinchoninic acid (BCA) protein assay kit, bovine serum albumin (BSA), a JC-1 mitochondrial membrane potential assay kit (C2006), crystal violet, N-acetyl-L-cysteine (NAC) (S0077), the Reactive Oxygen Species Assay Kit (S0033) and mouse anti-human GAPDH (AG019–1) were purchased from Beyotime Biotech (Shanghai, China). Monodansylcadaverine (MDC) (G0170) was purchased from Solarbio (Beijing China). Bafilomycin A1 (Baf-A1) (S1413) was purchased from Selleck (Houston, USA). Rabbit anti-human Bax (cat. D2E11), rabbit anti-human Bcl-2 (D55G8) (cat. 4223), rabbit anti-human cytochrome c (cat. 11940), rabbit anti-human cleaved caspase-3 (cat. 9664), rabbit anti-human MMP-9 (cat. 13667), the Cell Cycle Regulation Antibody Sampler Kit (cat. 9932), the Autophagy Antibody Sampler Kit (cat. 4445), rabbit anti-human SQSTM1/p62 (cat. 8025), rabbit anti-human AKT (cat. 4691), rabbit anti-human phospho-AKT (Ser 473) (cat. 4060), rabbit anti-human mTOR (cat. 2983), rabbit anti-human phospho-mTOR (Ser 2448) (cat. 5536), rabbit anti-human SAPK/JNK(cat. 9252) and rabbit anti-human phospho-SAPK/JNK (Thr183/Tyr185) (cat. 4668) were purchased from Cell Signaling Technology (Boston, MA, USA). Rabbit anti-human PCNA (3888) and rabbit anti-human Ki67 (4381) were purchased from Boster Biological Technology (Wuhan China). Rabbit anti-human Survivin (cat. 7642) and rabbit anti-mouse MMP2 (cat. 86607) were purchased from Abcam (MA, USA). Goat anti-rabbit (cat. 111–035-003) and goat anti-mouse secondary antibodies (cat. 115–035-003) were purchased from Jackson ImmunoResearch Laboratories (PA, USA).

### Cell culture

The OS cell lines MG63, 143B and HOS were purchased from American Type Culture Collection (ATCC; Manassas, VA, USA) and maintained in DMEM supplemented with 10% FBS, 100 u/mL penicillin and 100 μg/mL streptomycin. Cells were maintained in a humidified atmosphere with 5% CO2 at 37 °C.

### Cell viability assay

A CCK-8 assay was used to determine the viability of cells after the different treatments. Briefly, OS cells were seeded into 96-well plates at a density of 5,000 cells/well and incubated overnight for adherence. To determine the individual effects of CAP and DDP on OS cells, cells were incubated with various concentrations of CAP (0, 50, 100, 150, 200, 250 or 300 μM) or DDP (0, 8.3, 16.7, 25, 33.3, 50, 66.7, 83.3, 100, 116.6 or 133.3 μM) for 24 h. To determine the combined effects of CAP and DDP, OS cells were exposed to various combinations of relatively low concentrations of CAP (50, 100 and 100 μM) and various concentrations of DDP (0, 16.7, 33.3, 50, 66.7 μM) for 24 h. To assess the change in viability after the use of inhibitors, OS cells were preincubated with inhibitors (Baf-A1 or NAC) for 1 h before exposure to vehicle, CAP, DDP or CAP/DDP combination. Following the different treatments, 10 μL of CCK-8 was added to each well, and the plates were incubated for an additional hour. The plates with cells were subsequently placed in a microplate reader to detect the absorbance at 450 nm. Cell viability was calculated using the following formula: cell viability (%) = experimental group absorbance value / control group absorbance value X 100%.

### Combination index

The combined effect of DDP and CAP on OS cells was evaluated using the combination index (CI) as described previously [24]. The combined effect is classified as follows: CI < 1 is a synergistic effect; CI = 1 is an additive effect; and CI > 1 is an antagonistic effect. CI analysis was performed using Calcusyn Graphing Software (Biosoft, Inc., MO, USA).

### Cell apoptosis assay

Apoptosis was determined by flow cytometry using Annexin V-FITC/PI staining. Briefly, cells were seeded into 6-well plates (105 cells/well) and incubated overnight for adherence, and then the cells were exposed to CAP (100 μM), DDP (16.7 μM), or CAP and DDP combined for 24 h. After treatment, the cells were collected, washed twice with ice-cold phosphate-buffered saline (PBS), and stained with Annexin V-FITC and PI according to the manufacturer’s guidelines. The samples were then read on a flow cytometer (BD Biosciences, Franklin Lakes, NJ, USA). The distribution of viable (FITC-/PI-), early apoptotic (FITC+/ PI-), late apoptotic (FITC+/PI+) and necrotic (FITC-/PI+) cells was analyzed. Both early and late apoptotic cells were recorded as apoptotic cells, and the results are expressed as the percentage of total cells.

### Mitochondrial membrane potential assay

The mitochondrial membrane potential (Δψm) was detected using a JC-1 assay kit. Cells were seeded into 6-well plates at a density of 10^5^ cells/well and incubated overnight. After adherence, the cells were incubated with CAP (100 μM) or DDP (16.7 μM) alone or in combination for 24 h. Next, the cells were washed with PBS and incubated in medium containing 2 mM JC-1 at 37 °C for 20 min. After washing with ice-cold JC-1 buffer, the cells were directly observed under a fluorescence microscope. For flow cytometry assessments, after exposure to different treatments for 24 h, the cells were trypsinized, collected in medium containing JC-1 and then incubated at 37 °C for 20 min. After washing with ice-cold JC-1 buffer, the cells were analyzed using flow cytometry (BD Biosciences, Franklin Lakes, NJ, USA).

### Cell cycle assay

The cell cycle distribution was analyzed by flow cytometry with PI staining. Briefly, cells were seeded into 6-well plates at a density of 10^5^ cells/well and incubated overnight to allow adherence. Then, the cells were treated with CAP (100 μM), DDP (16.7 μM) or CAP in combination with DDP for another 24 h. The cells were trypsinized, collected and fixed in 70% ice-cold ethanol at − 20 °C overnight. Then, the cells were incubated with 10 mg/mL RNase and 50 μg/mL PI for 30 min. The cell cycle distribution was assessed using flow cytometry (BD Bioscience, Franklin Lakes, New Jersey).

### Cell invasion assay

The in vitro invasion activities of OS cells were evaluated using Transwell chambers with a 0.22-μm pore size (Corning, Inc.). The chambers was washed with serum-free medium, and then a layer of Matrigel was evenly plated on the surface of the membrane. Cells (10^5^ cells) were seeded into the upper chambers with 200 μL of treatment medium (control group, 100 μM CAP group, 16.7 μM DDP group and CAP/DDP combination group) and incubated in 24-well plates with 500 μL of medium containing 10% FBS in the lower chamber. After 24 h of treatment, non-invading cells were removed from the upper surfaces of the membranes. Invaded cells were stained with 1% crystal violet, and the cell numbers were counted under a microscope.

### Gelatin zymography assay

Cells were seeded into 6-well plates at a density of 10^5^ cells/well and incubated overnight to allow adherence. Then, the cells were treated with CAP (100 μM, serum-free medium), DDP (16.7 μM, serum-free medium), or CAP in combination with DDP for another 24 h. Next, the supernatant was collected, and the protein concentration was determined using the BCA Protein Assay Kit. Equal amounts of protein samples were separated by 10% SDS-PAGE containing 0.1% gelatin. After electrophoresis, the gels were washed twice with 2.5% Triton X-100 to remove SDS, and the following processes were performed according to the protocol for the MMP Zymography Assay Kit (Applygen Technologies Inc., Beijing, China). Finally, the gel was stained with Coomassie Blue and scanned by a gel documentation system (Bio-Rad, CYMML-EPMY-028, American).

### MDC staining

Autophagic vacuoles formed in the cells were detected by MDC staining. Cells (5 × 10^4^) were plated onto cover slips in 24-well plates and incubated overnight to allow adherence. Then, the cells were treated with vehicle or with CAP (100 μM) and DDP (16.7 μM) alone or in combination for another 24 h. Then, the cells were washed three times with PBS and incubated with MDC (50 μM) for 30 min at 37 °C. Next, the excess MDC was removed, and the cells on the cover slips were washed with PBS and fixed with 4% paraformaldehyde for 15 min. The autophagic vacuoles formed in the cells were analyzed by fluorescence microscopy with an excitation wavelength of 460–500 nm and an emission wavelength of 512–542 nm.

### Transmission electron microscopy (TEM)

Autophagy induction by the different treatments was evaluated by examining autophagosome formation using TEM. MG63 cells were seeded into a 6-well plate at a density of 10^5^ cells/wells overnight to allow adherence and then treated with CAP (100 μM) and DDP (16.7 μM) alone or in combination for another 24 h. Next, the cells were collected, fixed with 2.5% glutaraldehyde and 1% osmic acid, dehydrated with graded ethanol and acetone, embedded and sliced, and stained with 3% uranyl acetate-lead citrate. Finally, the cells were examined by TEM (JEM-1400 Plus, JEOL, Japan).

### Measurement of intracellular ROS

Intracellular ROS was detected using 2,7-dichlorofluorescin diacetate (DCFH-DA). Briefly, cells (10^5^ cells/well) were seeded into 6-well plates overnight to allow adherence and treated with various regimens for the indicated times. Then, the cells were washed with PBS three times and incubated in serum-free medium containing 10 μM DCFH-DA for 30 min at 37 °C in the dark. Next, the excess DCFH-DA was removed, and the cells were washed with PBS three times. The cells were observed immediately by fluorescence microscopy and then trypsinized, collected and detected by flow cytometry with an excitation wavelength of 488 nm and an emission wavelength of 530 nm.

### Western blot

After cells were treated with different regimens for the indicated times, they were lysed in RIPA lysis buffer containing PMSF and phosphatase inhibitors to extract the total intracellular proteins. Protein samples (30–50 μg/lane) were separated on an 8–12% gel by SDS-PAGE and transferred onto polyvinylidene fluoride (PVDF) membranes, which were blocked with either 5% skim milk or 5% BSA at room temperature for 1 h and then incubated with the corresponding primary antibodies (1:800) overnight at 4 °C. After the membranes were washed with Tris-buffered saline with Tween-20 (TBST), they were incubated with a secondary antibody for 1 h at 37 °C. The membranes were washed, and the reactive protein bands were detected with an enhanced chemiluminescence (ECL) detection system and developed on film.

### Xenograft tumor model

Twenty male nude mice (4 weeks old) were supplied by the Experimental Animal Center of Chongqing Medical University. All animal studies were approved by the Ethics Committee of Chongqing Medical University. The mice were housed with free access to a commercial diet and water under specific pathogen-free conditions. After the mice were acclimated for 1 week prior to study initiation, they were subcutaneously injected with 200 μL of sterile PBS containing a 143B cell suspension at a density of 10^6^ cells/mL. After the tumor volume reached 50 mm^3^, treatment was initiated. Twenty mice were randomized into the following 4 groups (5/group): (1) CAP group, mice were administered CAP (20 mg/kg) in 200 μL of PBS via oral gavage; (2) DDP group, mice were administered DDP (4 mg/kg) in 200 μL of 0.9% saline solution via intraperitoneal injection; (3) CAP/DDP combinational group, both CAP and DDP were administered according to the aforementioned regimens, respectively; and (4) Control group, mice were left untreated. All groups received their respective treatments every 3 days for a total of seven treatments. The body weights of the mice were measured every 6 days. The tumor volume was measured every 3 days after treatment according to the following formula: 1/2 x a^2^b (a is the short axis and b is the long axis of the tumor). Mice were sacrificed under anesthesia on day 27, and the xenograft tumors from each animal were weighed and analyzed. To investigate the nephrotoxicity induced by the different regimens, blood samples in each group were collected before sacrifice to evaluate serum creatinine and blood urea nitrogen (BUN) levels. Mice kidneys were rapidly dissected out and fixed in 10% formalin for histopathological studies. Furthermore, to investigate the lung metastasis of the subcutaneous xenotransplanted tumor under the regimens, lungs from the rats were dissected out and fixed in 10% formalin for histopathological studies. After fixation, tissues from xenograft tumors, kidneys and lungs were dehydrated in a graded series of ethanol and xylene, embedded in paraffin, cut into sections, and stained with hematoxylin and eosin (H&E). The H&E-stained sections were examined under a light microscope at a magnification of 200X.

### Immunohistochemistry (IHC)

IHC was performed to evaluate PCNA and Ki67 expression in xenograft tumor tissues. Briefly, the tumor tissues were separated, fixed with 4% paraformaldehyde, and embedded in paraffin. Next, the paraffin-embedded specimens were cut into serial sections (4 mm thick) by microtome. The tumor sections were blocked and immunostained with antibodies targeting Ki67 (1:200) and PCNA (1:200). Images were captured using a microscope, and PCNA and Ki67 expression were evaluated by counting the number of positive cells from 5 randomly selected fields in the residual viable tumor tissue among the necrotic areas under a light microscope at a magnification of 200X and 400X. Data are presented as the percentage of positive cells.

## Results

### Effects of CAP alone, DDP alone and their combination on the viability of OS cells

**Fig. 1:**
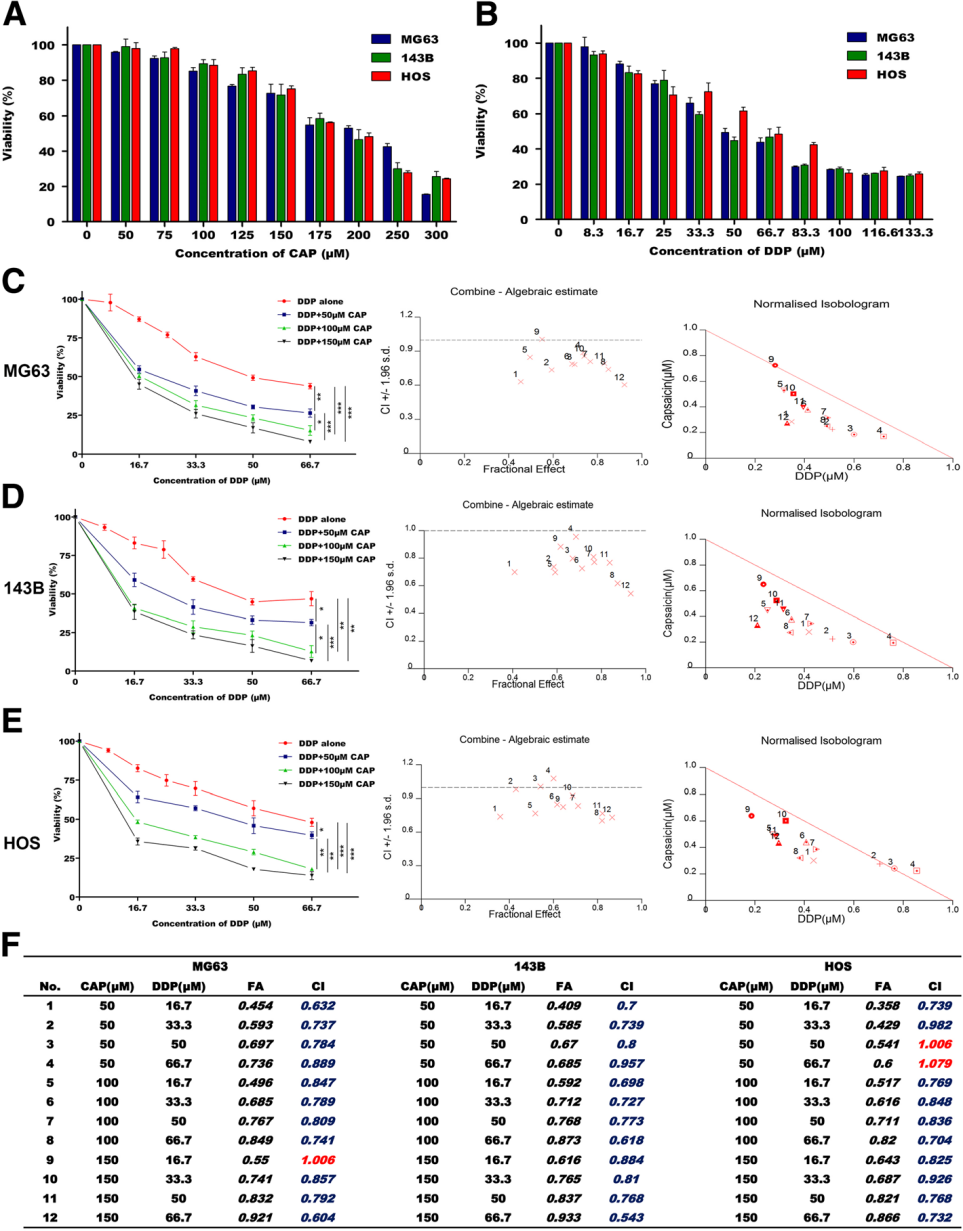
Effects of CAP and DDP on OS cell viability. Three different human OS cell lines (MG63, 143B and HOS) were treated with various concentrations of CAP (**a**) and DDP (**b**) for 24 h, and cell viability was measured using a CCK-8 assay. Three OS cell lines, MG63 (**c**), 143B (**d**) and HOS (**e**), were exposed to various combinations of concentrations of CAP and DDP for 24 h, and cell viability was measured using a CCK-8 assay. The combination index (CI) of CAP and DDP was calculated for the three OS cell lines (**f**). The data are presented as the mean ± SD; *p < 0.05, **p < 0.01 and ***p < 0.001 compared with untreated cells

The CCK-8 assay was used to examine the effects of CAP and DDP on the viability of three OS cell lines (MG63, 143B and HOS) in vitro. A wide range of concentrations of CAP and DDP were used to treat OS cells for 24 h. As shown in Fig. 1a-b, both CAP treatment alone and DDP treatment alone caused a concentration-dependent decrease in the viability of the three OS cell lines. After 24 h of treatment, the 50% inhibitory concentrations (IC50) of CAP in OS cells were 207.48 μM in MG63, 209.34 μM in 143B and 209.17 μM in HOS, respectively. In addition, the IC50 values of DDP in OS cells were 70.52 μM in MG63, 68.85 μM in 143B and 74.79 μM in HOS. Subsequently, a CCK-8 assay was used to evaluate whether a low-toxicity concentration of CAP can enhance the DDP-induced toxicity in OS cells. As our previous results indicated that relatively low concentrations of CAP exhibited mild cytotoxicity and could not induce significant apoptosis in OS cells, we performed viability tests of OS cells treated with low concentrations of CAP (50, 100, and 150 μM) in combination with DDP (16.7, 33.3, 50, and 66.7 μM) for 24 h to explore the effects of the combination. As shown in Fig. 1c-e, compared to DDP alone, the combination of CAP and DDP induced significantly higher cytotoxicity in the three OS cell lines. Furthermore, Chou-Talalay analysis was used to determine if CAP synergized with DDP to induce enhanced cytotoxicity [24]. A CI below 1 is an indication of synergism. The CI results for different combinations of CAP and DDP are summarized in Fig. 1f; the vast majority of combinations showed synergistic effects (CI < 1). These results indicated synergistic viability inhibition effects of low-toxicity concentrations of CAP and DDP on OS cells in vitro. Our primary goal in the present study was to determine whether low doses of CAP could sensitize human OS cells to DDP, with the aim of reducing the DDP dosage. Therefore, we selected 100 μM CAP and 16.7 μM DDP for our subsequent experiments, which both exhibited low toxicity when used separately and exhibited a significant synergistic effect when used in combination.

### Induction of apoptosis by CAP and DDP alone and in combination in OS cells

**Fig. 2:**
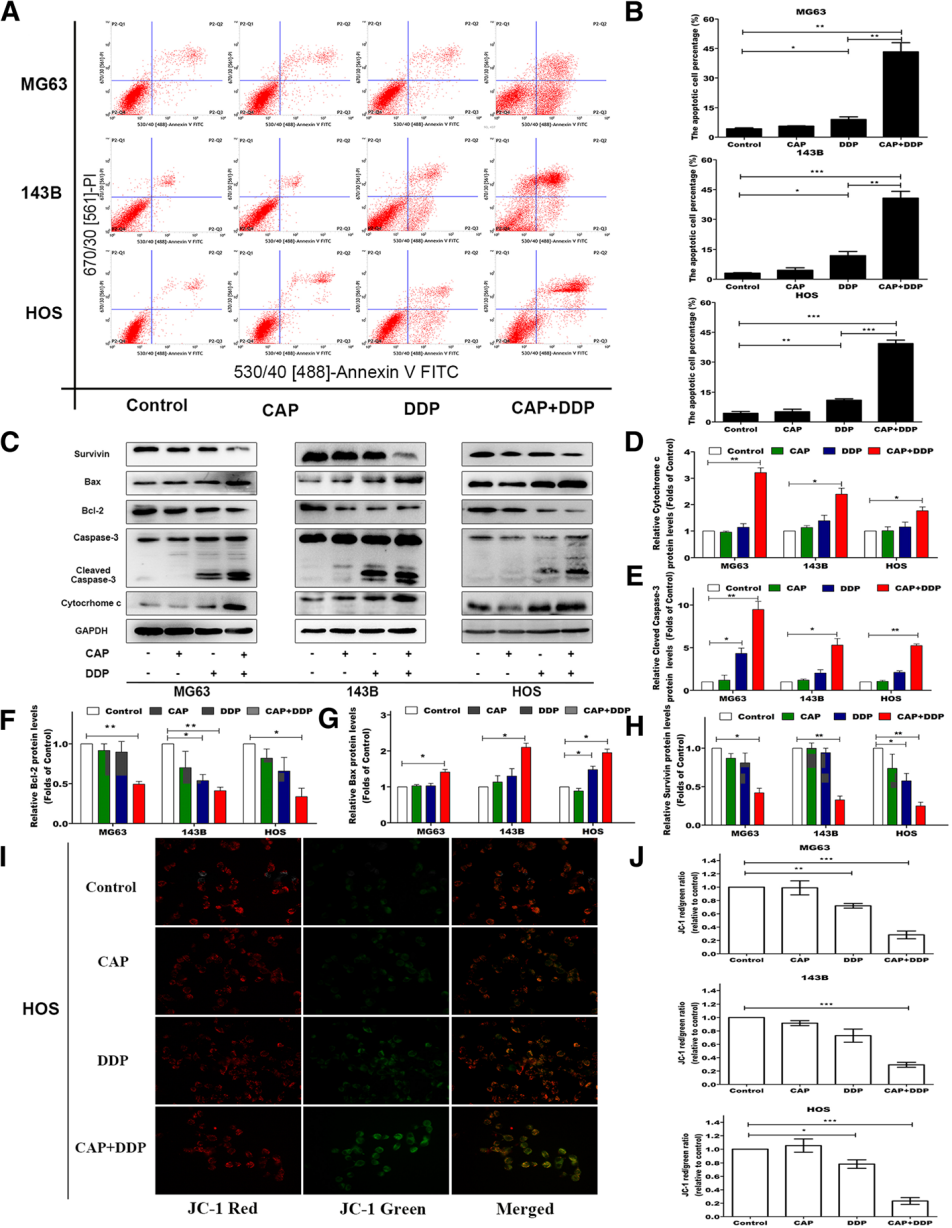
Induction of apoptosis by each treatment in OS cells. Cells were treated with CAP (100 μM) and DDP (16.7 μM) alone or in combination for 24 h. Apoptosis in MG63, 143B and HOS cells was determined by flow cytometry after staining with Annexin V-FITC/PI (**a**, **b**). The expression levels of cell apoptosis-related proteins were measured by Western blotting (**c**). Statistical analyses of cytochrome C (**d**), cleaved caspase-3 (**e**), Bcl-2 (**f**), Bax (**g**) and survivin (**h**) expression in the three OS cell lines in response to the different treatments. The effects of CAP and DDP on alterations of the mitochondrial membrane potential (Δψm) in HOS cells were determined by JC-1 staining via fluorescence microscopy (**i**). The Δψm (red/green) alteration in MG63, 143B and HOS cells was determined by flow cytometry after staining with JC-1 (**j**). The quantitative data are shown as the mean ± SD of 3 independent experiments; *p < 0.05, **p < 0.01 and ***p < 0.001 vs. the control (CAP -, DDP-)

To investigate whether the decrease in OS cell viability caused by CAP and DDP was related to induction of apoptosis, the apoptotic rate of OS cells treated with vehicle (as a control), 100 μM CAP and 16.7 μM DDP alone or in combination for 24 h was determined by flow cytometry using Annexin V-FITC/PI double staining. As shown in Fig. 2a-b, no obvious apoptotic effects were observed in the three OS cell lines upon treatment with CAP alone compared with the control group. However, treatment with DDP alone and the CAP/DDP combination both induced significant apoptosis (*p* < 0.05) compared with the control. In addition, the CAP/DDP combination treatment resulted in more significant apoptosis than DDP treatment alone in all three OS cell lines. To investigate the underlying mechanisms of the proapoptotic effect induced by CAP and DDP treatment, the expression levels of apoptosis-related proteins were assessed by Western blotting. After exposure to CAP, DDP alone and their combination for 24 h, we tested the expression of apoptosis-related proteins including cytochrome c, cleaved caspase-3, Bcl-2, Bax and survivin in the three OS cell lines. As shown in Fig. 2c-h, the proapoptotic proteins cytochrome c, cleaved caspase-3 and Bax were upregulated significantly, accompanied by a marked downregulation of the antiapoptotic proteins Bcl-2 and survivin after treatment with the combination of CAP and DDP compared with the levels in the control group. However, the apoptosis-related proteins showed no remarkable changes between the CAP-alone group and the control group. Interestingly, the DDP-alone treatment produced weak and unstable proapoptotic effects as detected by Western blot. However, considering the above mentioned results for the apoptosis assay by flow cytometry, we concluded that DDP alone could induce low levels of apoptosis in OS cells after 24 h of treatment. Considering the important role of mitochondria in cell apoptosis, we measured Δψm by a fluorescent JC-1 probe. During the apoptotic process, the mitochondrial membranes were disrupted with depolarization of Δψm, and mitochondrial depolarization was indicated by a decrease in the red to green fluorescence intensity ratio in the JC-1 assay. As shown in Fig. 2i, after treatment for 24 h with CAP, DDP and the CAP/DDP combination, HOS cells were stained with JC-1, and the results showed that the DDP- and CAP/DDP-treated cells exhibited a decrease in red fluorescence and an increase in green fluorescence. These changes were more prominent after the CAP and DDP combination treatment. However, there was no change in JC-1 fluorescence in the CAP-alone group compared with that in the control. Figure 2j shows the assay of JC-1 by flow cytometry. Both DDP alone and CAP/DDP treatment reduced the red/green fluorescence ratio in the three OS cell lines. However, the CAP/DDP combination resulted in more significant reductions.

### Cell cycle arrest induced by CAP and DDP alone or in combination in OS cells

**Fig. 3:**
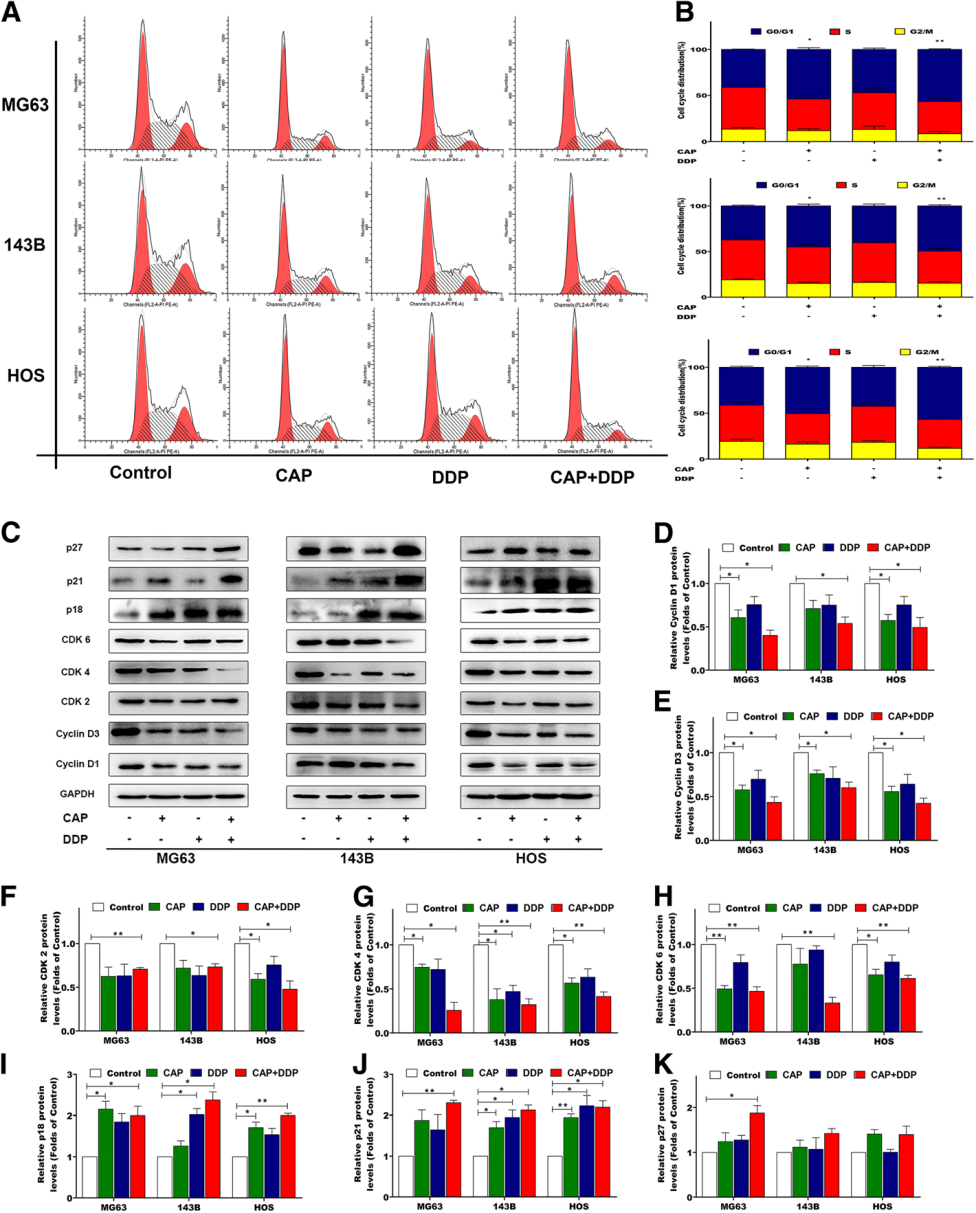
Effects of each treatment on the cell cycle distribution in OS cells. Three OS cell lines (MG63, 143B and HOS) were treated with CAP (100 μM) and DDP (16.7 μM) alone or in combination for 24 h. Then, the cells were stained with PI and analyzed by flow cytometry (**a**, **b**). The expression levels of cell cycle-related proteins were measured by Western blotting (**c**). Statistical analyses of cyclin D1 (**d**), cyclin D3 (**e**), CDK2 (**f**), CDK4 (**g**), CDK6 (**h**), p18 (**i**), p21 (**j**) and p27 (**k**) in the three OS cell lines. The quantitative data are shown as the mean ± SD of 3 independent experiments; *p < 0.05, **p < 0.01 and ***p < 0.001 vs. the control (CAP-, DDP-)

To investigate the effects of CAP and DDP on the cell cycle, the cell cycle distributions after treatment were determined by flow cytometry. Three OS cell lines were treated with CAP (100 μM) and DDP (16.7 μM) alone or in combination for 24 h; the results in Fig. 3a-b show that CAP alone or in combination with DDP resulted in an increased percentage of arrest at G0/G1 phase compared with that in the control group. However, the application of DDP alone had no effects on cell cycle progression in all OS cells lines. To explore the potential molecular mechanism of G0/G1 cell cycle arrest, the expression levels of cell cycle-related proteins were determined by Western blot analysis. As shown in Fig. 3c-k, CAP alone or in combination with DDP caused decreases in Cyclin D1, Cyclin D3, CDK2, CDK4 and CDK6 but increases in p18, p21 and p27 in the three OS cell lines, whereas the alterations of the cell cycle-related proteins were not significant when the DDP-alone group was compared with the control group.

### Effect of CAP and DDP alone or in combination on the invasive ability of OS cells

**Fig. 4:**
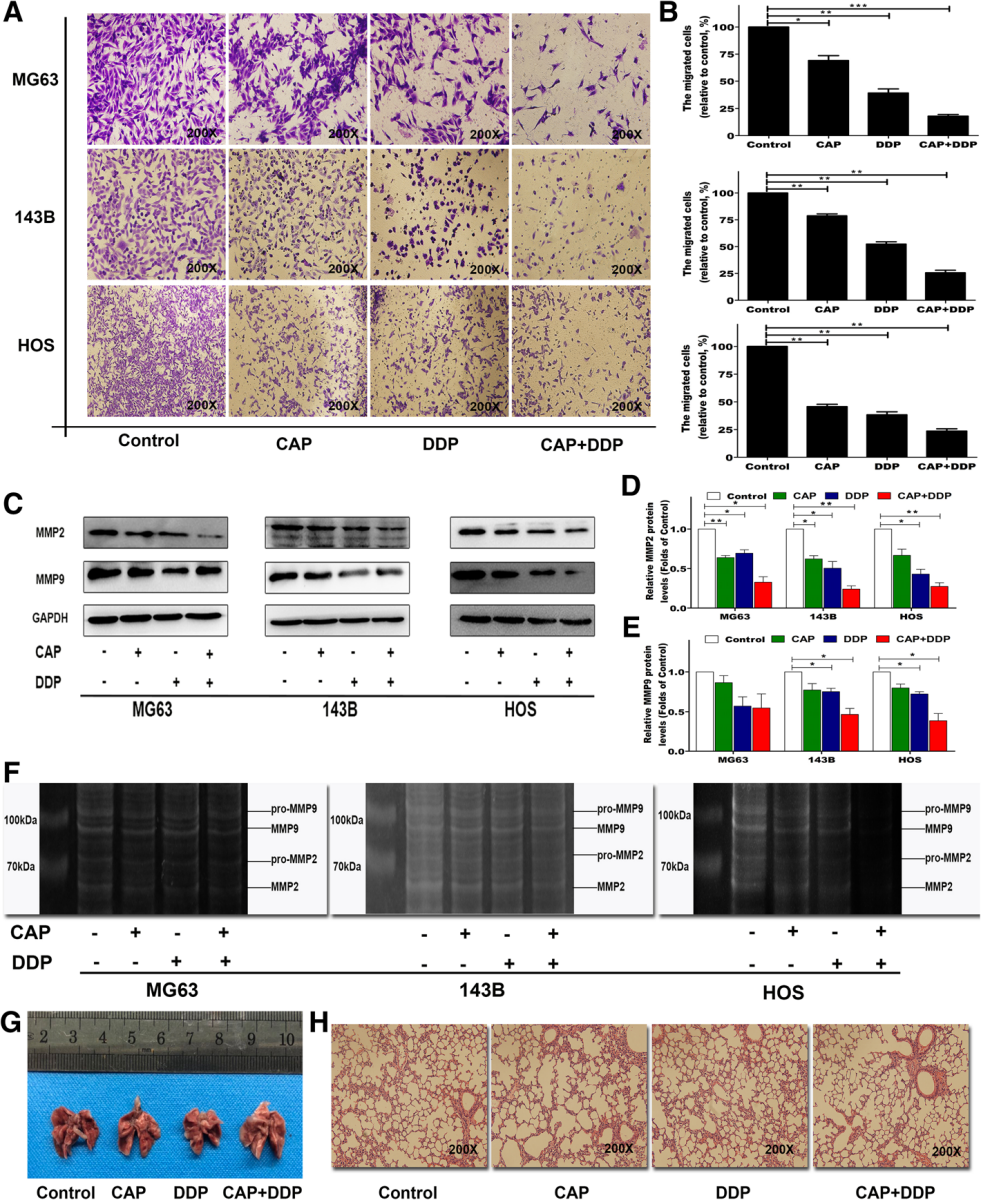
Effects of each treatment on the invasive ability of OS cells. Cell invasion by MG63, 143B and HOS cells was assessed via Transwell invasion assays; crystal violet staining of the cells that passed through the polycarbonate membrane after treatment with CAP (100 μM) and DDP (16.7 μM) alone or in combination for 24 h was observed by microscopy (**a**). The invasive abilities were quantified by counting the number of cells, and the percentage of the treated group compared to the control group (CAP-, DDP-) is presented as the mean ± SD of 3 independent experiments, *p < 0.05, **p < 0.01 and ***p < 0.001 vs. the control (**b**). The expression levels of cell invasion-related proteins were measured by Western blotting (**c**). Statistical analyses of MMP-2 (**d**) and MMP-9 (**e**) in the three OS cell lines. The MMP activity of MMP-2 and MMP-9 was analyzed by gelatin zymography (**f**). After the last treatment, the mice were sacrificed, and the lungs were collected from the different treatment groups to assess distant metastatic foci (**g**). Representative histological profiles of lungs after different treatments as detected by H&E staining (**h**). The quantitative data are shown as the mean ± SD of 3 independent experiments; *p < 0.05 and **p < 0.01 vs. the control (CAP-, DDP-)

Transwell analyses were performed to evaluate the inhibitory effect of CAP and DDP used individually or in combination on the invasive ability of OS cells. As shown in Fig. 4a-b, after 24 h of treatment, the application of CAP and DDP alone or in combination inhibited the invasive abilities of OS cells significantly. In addition, the CAP/DDP combination group showed the greatest inhibitory effects on OS cell invasion. To investigate whether the inhibition of OS cell invasion was associated with the regulation of matrix metalloproteinases (MMPs), the expression of the invasion-related proteins MMP-2 and MMP-9 was determined by Western blotting, and the activation of MMP-2 and MMP-9 was determined by gelatin zymography assays. As shown in Fig. 4c-e, the expression of MMP-2 and MMP-9 was decreased in the groups treated with CAP and DDP alone or in combination compared with that in the control group. Moreover, the combination group showed the greatest reduction in these two proteins in all three OS cell lines. Furthermore, as shown in Fig. 4f, the ability of MMP-2 and MMP-9 to degrade gelatin was significantly reduced after treatment with CAP and DDP alone or in combination, and the combination of CAP and DDP showed the most significant effect. To investigate the effects of CAP and DDP on the invasive ability of OS cells in vivo, we assessed lung metastasis after the different treatments. However, no lung metastatic foci were found in any of the groups (Fig. 4g, h).

### Autophagy induced by the treatment of CAP and DDP alone or their combination in OS cells

**Fig. 5:**
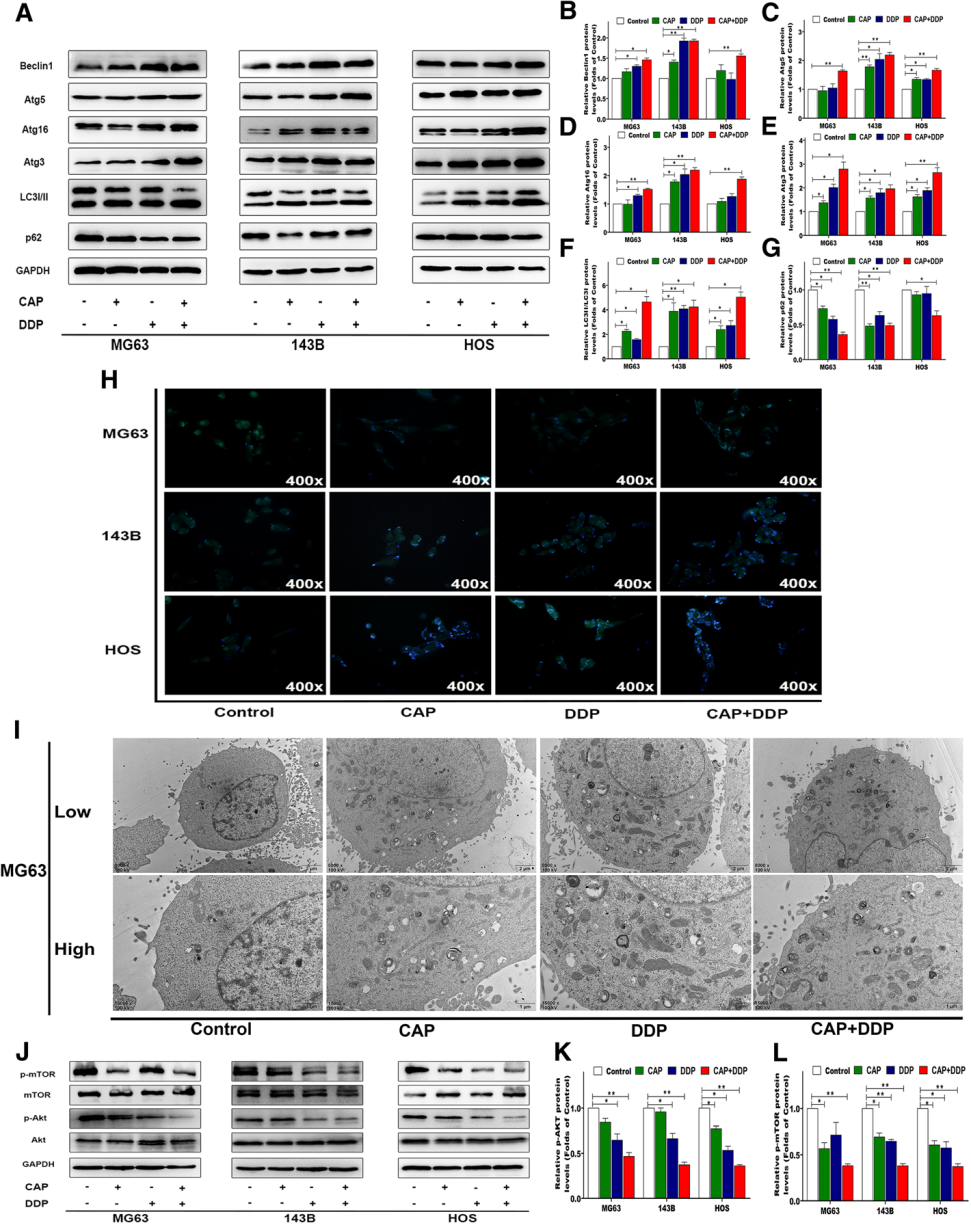
Autophagy induction by each treatment in OS cells. MG63, 143B and HOS cells were treated with CAP (100 μM) and DDP (16.7 μM) alone or in combination for 24 h. The expression levels of cell autophagy-related proteins were measured by Western blotting (**a**). Statistical analyses of beclin1 (**b**), Atg5 (**c**), Atg16 (**d**), Atg3 (**e**), LC3II/LC3I (**f**) and P62 (**g**) in the three OS cell lines. Following each treatment, acidic vacuoles in the three OS cell lines were stained with MDC and observed under a fluorescence microscope (**h**). Transmission electron microscopy (TEM) was utilized to evaluate the changes in autophagosomes in MG63 cells after each treatment (**i**). The expression levels of critical proteins involved in the AKT/mTOR signaling pathway were measured by Western blotting (**j**). Statistical analyses of p-AKT (k) and p-mTOR (l) in the three OS cell lines. The quantitative data are shown as the mean ± SD of 3 independent experiments; *p < 0.05 and **p < 0.01 vs. the control (CAP-, DDP-)

Previous studies have demonstrated that both CAP and DDP can induce autophagy in cancerous cells in vitro. To explore whether the combination of CAP and DDP can trigger autophagy in OS cells, the expression of autophagy-associated proteins after treatment for 24 h was determined by Western blot. Beclin1 and various Atgs play an essential role in autophagy initiation. Figure 5a-g shows that the CAP/DDP combination could upregulate proteins including Beclin1, Atg3, Atg16 and Atg5 in OS cells. The conversion of LC3-I to LC3-II is considered the hallmark of mammalian autophagy. Our findings showed that the conversion of LC3B-I to LC3B-II was significantly increased in the CAP/DDP combination group compared with that in the control group. Next, we also used MDC staining to detect autophagic vacuoles. As shown in Fig. 5h, the accumulation of MDC-labeled vacuoles in the cytoplasm in the three OS cell lines was significantly higher in the CAP/DDP combination group than in the control group. Furthermore, we observed autophagosome formation in MG63 cells via TEM. As shown in Fig. 5i, the number of autophagic vacuoles was obviously greater in the CAP/DDP combination group than in the control group. The ROS/AKT/mTOR signaling pathway plays a crucial role in mediating cellular autophagy, and the activation of mTOR suppresses autophagy. As shown in Fig. 5j-k, the CAP/DDP combination treatment caused a significant decrease in p-AKT in the three OS cell lines and further resulted in a reduction of p-mTOR.

**Fig. 6:**
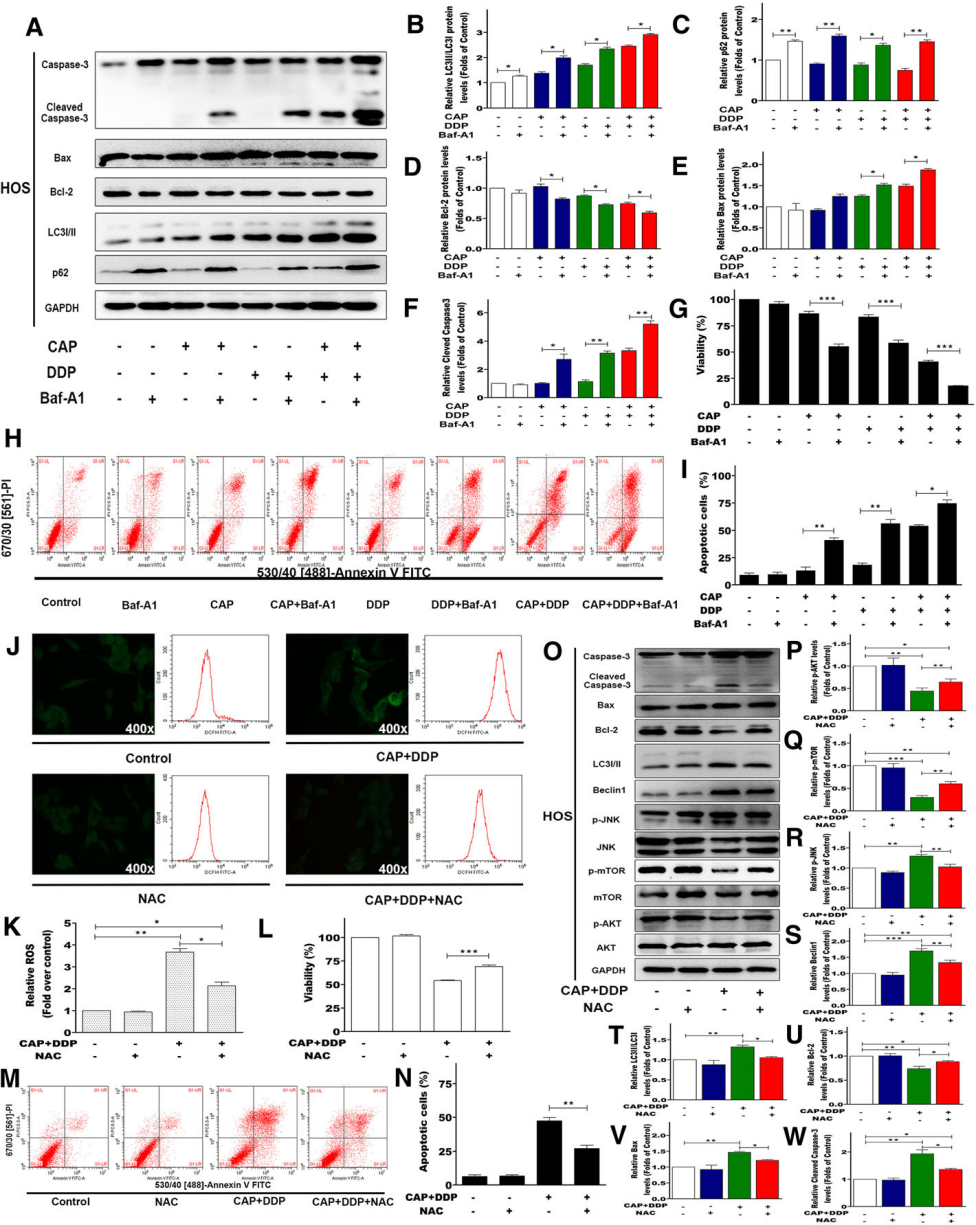
The role of autophagy induction in the response to each treatment in OS cells. HOS cells were pretreated with Baf-A1 (100 nM) for 2 h prior to treatment with CAP (100 μM) and DDP (16.7 μM) alone or in combination for 24 h. The expression levels of autophagy-related and apoptosis-related proteins were measured by Western blotting (**a**). Statistical analyses of LC3II/LC3I (**b**), p62 (**c**), Bcl-2 (**d**), Bax (**e**), and cleaved caspase-3 (**f**) in HOS cells. The relative cell viability (%) was determined using a CCK-8 assay (**g**). Apoptosis in HOS cells was determined by flow cytometry after staining with Annexin V-FITC/PI (**h**, **i**). The quantitative data are shown as the mean ± SD of 3 independent experiments; *p < 0.05, **p < 0.01 and ***p < 0.001 vs. the control (CAP-, DDP-, Baf-A1-). The role of reactive oxygen species (ROS) generation in HOS cells after CAP/DDP combination treatment was assessed. HOS cells were pretreated with NAC (5 mM) for 2 h prior to cotreatment with CAP (100 μM) and DDP (16.7 μM) for another 24 h. The levels of cellular ROS were determined by fluorescence microscopy and flow cytometry after DCFH-DA (10 mM) staining (**j**, **k**). The relative cell viabilities (%) were determined using the CCK-8 assay (**l**). The apoptosis percentages were determined by flow cytometry after staining with Annexin V-FITC/PI (M, N). After ROS scavenging by NAC, the expression levels of proteins involved in autophagy induction, apoptosis, the ROS/JNK signaling pathway and the AKT/mTOR signaling pathway were measured by Western blotting (**o**). Statistical analyses of p-AKT (**p**), p-mTOR (**q**), p-JNK (**r**), beclin1 (**s**), LC3II/LC3I (**t**), Bcl-2 (**u**), Bax (**v**), and cleaved caspase-3 (w) in HOS cells. The quantitative data are shown as the mean ± SD of 3 independent experiments; *p < 0.05, **p < 0.01 and ***p < 0.001 vs. the control (CAP+DDP-, NAC-)

As the induction of autophagy could either promote cell survival or contribute to cell death, to investigate whether the autophagy induction by CAP and DDP is prosurvival or prodeath, Baf-A1, an autophagy inhibitor, was used to pretreat HOS cells before CAP or DDP treatment. As shown in Fig. 6a-f, with pretreatment with Baf-A1, the expression of LC3II/LC3I and p62 levels in the CAP- and DDP-alone or CAP/DDP combination groups were all elevated significantly, indicating effective blockage of autophagy. Correspondingly, the expression levels of the proapoptotic proteins cleaved caspase-3 and Bax were upregulated, and the antiapoptotic protein Bcl-2 was downregulated after autophagy blockage. Furthermore, our results, as shown in Fig. 6g-i, also suggested that pretreatment with Baf-A1 could enhance the inhibitory effects of CAP and DDP on HOS cell viability and promote the apoptotic effects induced by CAP and DDP. Taken together, these results indicated that the autophagy induction by CAP and DDP treatment may be prosurvival in OS cells.

### Elevation of ROS generation in HOS cells by the combination of CAP and DDP is involved in apoptosis and autophagy by mediating the AKT/mTOR and JNK signaling pathway

ROS are important regulators in various pathways, including apoptosis and autophagy. To investigate whether treatment with CAP and DDP could induce ROS generation in HOS cells, DCFH-DA was used to assess cellular ROS levels. As shown in Fig. 6j-k, HOS cells treated with the CAP/DDP combination exhibited a significant increase in ROS generation compared with the control. However, the increase in ROS production was effectively inhibited in HOS cells after pretreatment with NAC, an ROS scavenger. We further determined whether pretreatment with NAC could protect HOS cells from the cytotoxic effects of CAP/DDP combination treatment. As shown in Fig. 6l-n, cells pretreated with NAC followed by CAP/DDP combination treatment resulted in an increase in the viability of HOS cells compared with cells treated with the combination of CAP and DDP. Moreover, the apoptosis assay also showed that NAC significantly attenuated the apoptotic effects induced by CAP/DDP combination treatment in HOS cells. As ROS is a well-known inducer of both apoptosis and autophagy, we next investigated the role of ROS in apoptosis and autophagy and the potential signaling pathways. As shown in Fig. 6o-w, NAC attenuated CAP/DDP-induced apoptosis by downregulating the proapoptotic protein Bax, consistent with the upregulation of the antiapoptotic protein Bcl-2 and ultimately resulting in decreased cleaved caspase-3 levels. In addition, autophagy-related markers, including the LC3II/LC3I and Beclin1 proteins, were also reduced significantly by pretreatment with NAC. ROS have been demonstrated to be inducers or mediators of the activation of the JNK signaling pathway, and our results also showed that pretreatment with NAC significantly decreased the phosphorylation of JNK induced by CAP/DDP combination treatment. Furthermore, ROS have also been reported to play an important role in mediating the ROS/AKT/mTOR signaling pathway, which is important in regulating cell apoptosis and autophagy. Our current results showed that NAC could partially reverse the reductions in p-AKT and p-mTOR induced by CAP/DDP combination treatment. Taken together, the results suggested that the apoptosis and autophagy induced by the combination of CAP and DDP in HOS cells could be mediated by the elevation of ROS through the ROS/JNK and ROS/AKT/mTOR signaling pathways.

### Inhibitory effects of CAP and DDP on tumor growth in a xenograft model of human OS

**Fig. 7:**
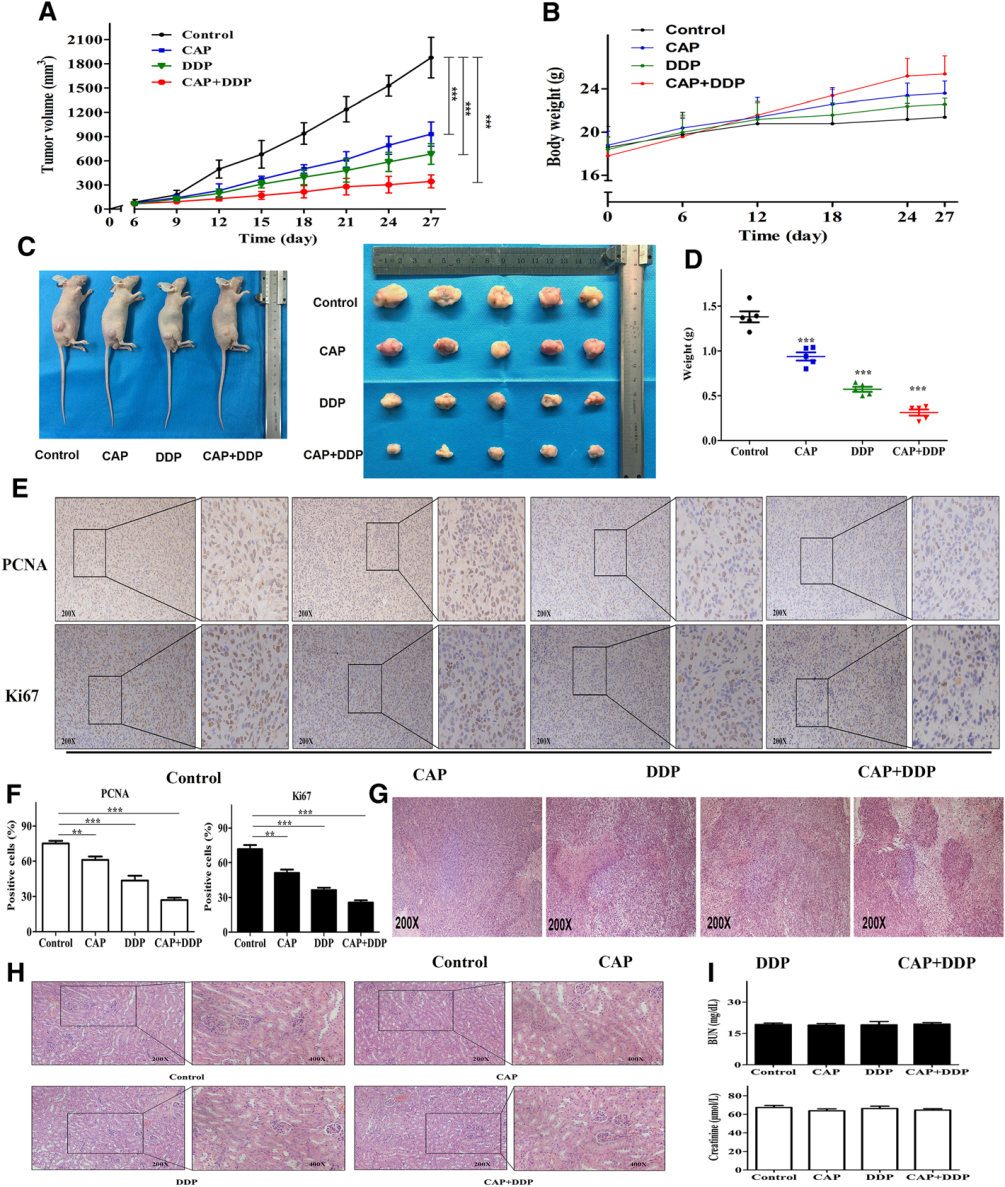
Effects of each treatment on the growth of human OS xenograft tumors. Nude mice bearing 143 human OS xenograft tumors were treated with CAP (20 mg/kg) and DDP (4 mg/kg) alone or in combination. The volumes of the xenograft tumors were measured at the indicated time points (**a**). The weight of each nude mouse was measured at the indicated time points (**b**). After the last treatment, the mice were sacrificed, and representative images of the subcutaneous tumor xenografts in the nude mice and the morphology of the tumors are presented (**c**). Tumors were collected, and the tumor weights were measured and compared (**d**). Xenograft tumors were sectioned and stained with PCNA and Ki67 via IHC (**e**). Statistical analyses of the expression of PCNA and Ki67 in different groups (**f**). Tumors were sectioned and stained with H&E, and representative histopathological images are presented (**g**). Effects of the different treatments on renal histology. Representative histological profiles of kidneys after the different treatments were detected by H&E staining (**h**). Effects of the different treatments on BUN and creatinine levels in mice (**i**). The quantitative data are shown as the mean ± SD of 5 independent experiments; *p < 0.05, **p < 0.01 and ***p < 0.001 vs. the control (CAP-, DDP-)

Based on the inhibitory effects of CAP and DDP treatment on OS cells in vitro, we investigated whether similar inhibitory effects could occur in tumor growth in a xenograft model. Nude mice were subcutaneously implanted with 143B cells, and the tumor volumes were measured every 3 days. Tumor-bearing mice were randomly divided into four groups: a control group (injections of solvent alone), a CAP-alone treatment group, a DDP-alone treatment group and a group treated with a combination of CAP and DDP. As shown in Fig. 7a, all treated groups, including those with CAP and DDP treatment alone or combination treatment, exhibited a significant reduction of tumor volume compared with that in the control group. However, the CAP/DDP combination treatment group showed the greatest inhibitory effects on tumor volume. No significant difference in body weight was observed during the experimental period between the control and treated groups (Fig. 7b). At the end of the experiment, the tumor weight measurements showed that the three treated groups exhibited significantly decreased tumor weights compared with the tumor weights in the control group. In addition, the combined treatment resulted in tumors that were smaller than those treated with either CAP or DDP alone (Fig. 7c, d). We also evaluated the expression of proliferative markers, including PCNA and Ki67, to further validate the inhibitory effects of CAP and DDP on OS tumor growth. The expression levels of both PCNA and Ki67 in the tumor specimens from the three treated groups were lower than those in the control group. Similarly, the combined treatment group showed the lowest expression of these two indicators among the three treated groups (Fig. 7e, f). Specimens from the xenograft tumors were stained with H&E after mouse sacrifice, as tumor necrosis after chemotherapy is a crucial criterion for evaluating the response to treatment in OS. Our results showed that tumor specimens from the treatment groups (CAP alone, DDP alone, and CAP/DDP combination) showed varying grades of necrosis, and the combination treatment with CAP and DDP was associated with the most severe necrosis (Fig. 7g). Changes in renal histology were used to evaluate the nephrotoxic effects of the different treatments, and the results showed that all kidney sections from the control and treatment groups had a normal histopathological appearance. No histopathological changes in the kidney such as necrosis of tubular cells, hyaline cast formation, mononuclear infiltration and glomerular necrosis were observed (Fig. 7h). In addition, there was no significant alteration of creatinine or BUN levels in mice treated with CAP and DDP alone or in combination compared with those in the control group (Fig. 7i). Taken together, these findings indicated that all of the treatments with CAP and DDP alone or in combination significantly inhibited OS tumor growth in the xenograft model without significant nephrotoxicity. Furthermore, the combined treatment with CAP and DDP exhibited the most prominent therapeutic effect.

## Discussion

DDP is used as the first-line chemotherapeutic agent in the treatment of OS, but its efficacy is limited by the development of resistance and normal tissue toxicity. Some conventional agents including methotrexate, doxorubicin and etoposide may be combined with DDP to improve efficacy and minimize toxicity, but multidrug resistance to these drugs should not be overlooked [6]. Therefore, it is very important to seek a potential agent that can enhance the sensitivity of OS to conventional chemotherapeutic drugs. Due to their chemoprotective and anticancer characteristics, phytochemicals alone or together with other traditional chemotherapeutic agents have been introduced in the treatment of various cancers [25, 26]. Previous studies have reported the use of phytochemicals with anticancer activities in combination with DDP to achieve greater efficacy for OS chemotherapy [18, 27]. CAP is the most abundant and pungent component in a variety of hot peppers, and its anticancer effects have garnered increasing attention recently [22]. Our previous study revealed that CAP inhibits the proliferation and colony formation of OS cells in a dose-dependent manner. However, apoptotic effects were only observed when the OS cells were exposed to relatively high concentrations of CAP (starting at 250 μM) [23]. Furthermore, Jung et al. [28] demonstrated that CAP has protective effects against cisplatin-induced renal dysfunction. These results suggest that CAP may exert therapeutic benefits as an adjunct to conventional chemotherapies but not as an independent anticancer agent. Therefore, we performed this study to assess the effects of using CAP combined with DDP on OS. In the present study, both CAP alone and DDP alone were validated to have inhibitory effects on OS cells in a dose-dependent manner. More importantly, our results further demonstrated that combination regimens with CAP and DDP at subtoxic concentrations showed significant synergistic anticancer effects (CI < 1) on OS cells. These findings suggested that CAP might be used as a chemotherapeutic agent to enhance anticancer effects in OS cells when combined with DDP.

Many chemotherapeutic agents primarily exhibit their anticancer effects by inducing apoptosis in cancer cells [29]. However, the apoptosis induction efficacy of conventional agents gradually decreases due to chemoresistance [30]. DDP resistance is mainly caused by inhibition of apoptosis of cancer cells [31]. Therefore, an effective adjuvant to DDP-based chemotherapy should sensitize cancer cells to DDP-induced apoptosis. In the present study, subtoxic concentrations of CAP (100 μM) and DDP (16.7 μM) were selected to explore their combined effects on OS cells. The results showed that either CAP or DDP could induce indeterminate apoptosis in the three OS cells. However, the CAP/DDP combination induced apoptosis significantly. These results indicated that CAP is a potential candidate for strengthening the DDP-induced apoptotic effect in OS cells. The induction of apoptosis involves two classical pathways: the death receptor pathway (extrinsic pathway) and the mitochondrial pathway (intrinsic pathway) [32]. The metabolic activities of mitochondria in cancer cells are higher than those in normal cells. Thus, the mitochondrial apoptotic pathway is considered an important target in cancer therapy [33]. In the mitochondrial apoptotic pathway, loss of Δψm leads to an increase in mitochondrial membrane permeability, which leads to the release of cytochrome c from the mitochondria to the cytoplasm. In the cytoplasm, cytochrome c initiates the activation of caspases and eventually induces cell apoptosis. The release of cytochrome c is prevented by the Bcl-2 protein. In the present study, the Δψm in OS cells was decreased significantly in the CAP/DDP group compared with that in the control group. Furthermore, we observed a decrease in the antiapoptotic protein Bcl-2 with an increase in the proapoptotic protein Bax, and these changes were accompanied by an increase in cytochrome c in the CAP/DDP combination group. Furthermore, there were no significant changes when the control group was compared with either the CAP-alone or DDP-alone treatment groups. These results demonstrated that combination treatment using low concentrations of CAP and DDP could effectively induce apoptosis in OS cells through the mitochondrial apoptotic pathway.

Cell cycle progression is strictly supervised by a number of checkpoints that ensure the accuracy and integrity of DNA replication. When DNA is damaged by certain factors, these checkpoints initiate cell cycle arrest followed by activation of repair systems or induction of apoptosis. Evasion of cell cycle arrest is most frequently observed in tumor development [34, 35]. One of the anticancer mechanisms of chemotherapeutic agents is the induction of cell cycle arrest in cancer cells [36, 37]. It has been reported that the combination of other agents with DDP significantly induces various levels of cell cycle arrest in OS cells [38, 39]. One of the major anticancer effects of CAP is to induce cell cycle arrest in OS cells [23]. Therefore, we investigated the cell distribution in cell cycle phases after treatment with CAP and DDP alone or in combination. Our results revealed that CAP alone or in combination with DDP could induce G0/G1 cell cycle arrest in OS cells. However, the low concentration of DDP applied in the present study failed to inhibit cell cycle progression in OS cells. Moreover, the cell cycle-related proteins in OS cells also showed corresponding alterations. Although the combination of CAP and DDP inhibited the cell cycle transition by inducing G0/G1 arrest, the phenomenon and tendency were similar to those in the CAP-alone group. Our present results indicated that although synergistic suppression of cell viability was observed, the lower-concentration combination of CAP and DDP might not be able exert a synergistic effect on modulation of cell cycle progression.

Local invasion and distant metastases are frequently observed in OS patients, which is the major obstacle in the comprehensive treatment of OS [40]. Emerging evidence suggests that MMPs play important roles in tumor invasion and metastasis and are abundantly expressed in various malignant tumors [41]. Among the MMPs, MMP-2 and MMP-9 are essential for the initiation of migration and invasion [42]. Previous studies have reported that CAP and DDP alone can suppress malignant cell invasion, which is associated with the downregulation of MMP-2 and MMP-9 [38, 43, 44]. Thus, we performed Transwell migration assays of three OS cell lines to determine the effects of CAP and DDP as well as their combination on OS migration. Our results indicated that using CAP and DDP individually inhibited the invasion of OS cells, and the CAP/DDP combination treatment showed the greatest inhibitory effects. Furthermore, the inhibitory effects on migration and invasion were correlated with reduced expression and downregulation of the activation of MMP-2 and MMP-9. In conclusion, our results demonstrated that the use of CAP could significantly enhance the antimigratory effect of DDP on OS cells in vitro by suppressing the expression of MMP-2 and MMP-9.

Autophagy is an evolutionarily conserved process in which cytoplasmic materials and organelles are sequestered into autophagosomes and further degraded in the autophagolysosomes for recycling to maintain cellular homeostasis [45]. Autophagy can be simulated by various factors, including starvation, growth factor deprivation, hypoxia and exposure to cytotoxic compounds. Substantial evidence has demonstrated the crucial role of autophagy in cancer initiation, differentiation, proliferation, metastasis and chemoresistance. Moreover, multiple studies have attempted to elucidate the paradoxical role of autophagy in cancer, which has provided a better understanding of autophagy in tumorigenesis and has established autophagy as a target for cancer treatment [46]. A large number of phytochemicals and conventional chemotherapeutics have been shown to induce autophagy in various cancerous cells [47, 48]. In the present study, we observed that the use of CAP or DDP alone could induce autophagy in OS cell lines via MDC staining and TEM. The LC3II/LC3I ratio, an indicator of autophagy, was significantly increased. Furthermore, we also observed an increase in the levels of various proteins involved in autophagosome formation, including Beclin1, Atg3, Atg5, and Atg16. Our present results revealed that using CAP or DDP alone could induce autophagy in OS cells and that the combination of CAP and DDP induced more significant autophagy than either agent alone. The AKT/mTOR signaling pathway plays crucial roles in many cellular functions such as cell growth, apoptosis and differentiation. Aberrant activation of the AKT/mTOR pathway is widely observed in various malignant tumors, which accelerates proliferation, increases resistance to apoptosis and promotes invasion and metastasis [49]. Moreover, the AKT/mTOR signaling pathway has been reported to negatively regulate autophagy, and the inhibition of mTOR activation promotes autophagy [50]. The present results showed that the expression of p-AKT and p-mTOR were significantly decreased after exposure to CAP alone, DDP alone or the CAP/DDP combined treatment, indicating suppression of the AKT/mTOR signaling pathway in OS cells. Furthermore, the inhibition was more significant with the combination of CAP and DDP than either CAP or DDP alone. In conclusion, our present results demonstrated that CAP and DDP or their combination induced autophagy in OS cells, and this process was negatively mediated by the ROS/AKT/mTOR signaling pathway.

The present findings are consistent with those of previous studies showing that many anticancer agents can induce autophagy in OS cells. However, the effects of autophagy in determining cancer fate, either by promoting cell survival or inducing cell death, are still controversial. Moreover, the precise crosstalk between autophagy and apoptosis has not yet been elucidated. To further investigate the promotional or antagonistic role of autophagy in mediating apoptosis in OS cells after treatment with CAP and DDP alone or in combination, we assessed cellular viability and apoptosis as well as the expression of related proteins after blocking autophagy with Baf-A1, a late-stage autophagy inhibitor that prevents the fusion of autophagosomes with lysosomes by inhibiting V-ATPase-dependent acidification [46]. Our results showed that pretreatment with Baf-A1 decreased HOS cell viability and increased apoptosis compared with those in the groups that were not treated with Baf-A1. Western blots also showed that the use of Baf-A1 significantly increased the expression of proapoptotic proteins such as Bax, cytochrome c and cleaved caspase-3 and decreased the expression of the antiapoptotic protein Bcl-2 compared with treatment with CAP alone, DDP alone and the combination. Taken together, these findings strongly indicate that the induction of autophagy might play a prosurvival role in OS cells under the three different treatments in the current study.

ROS serve as an important molecular signal in various vital cellular processes, including proliferation, differentiation and apoptosis [51]. Numerous lines of evidence have also demonstrated the critical role of ROS in the initiation and mediation of autophagy [52]. Thus, we explored the role of ROS-mediated autophagy and apoptosis in HOS cells exposed to the CAP/DDP combination treatment. We observed a significant elevation of intracellular ROS after CAP/DDP combination treatment. However, in cells pretreated with NAC, a general ROS scavenger, the ROS elevation induced by CAP/DDP treatment was effectively reversed. Furthermore, the viability of HOS cells was preserved, and CAP/DDP-induced apoptosis was also partially suppressed. As the activation of ROS/JNK has been suggested to effectively induce apoptosis in cancerous cells [53], to further explore the underlying mechanisms of ROS elevation in OS cells after treatment with the CAP/DDP combination, we assessed the key protein alterations involved in apoptosis, autophagy and the associated signaling pathways, including AKT/mTOR and ROS/JNK, with or without pretreatment with the ROS scavenger NAC. The present results showed that NAC modulated the ratio of Bax/Bcl-2 by downregulating Bax and upregulating Bcl-2, which resulted in decreased expression of cleaved caspase-3. These results were consistent with those obtained by flow cytometry, which showed that NAC inhibited the apoptosis induced by the CAP/DDP combination. In addition, NAC also decreased the expression of beclin1 and the conversion of LC3I to LC3II, which indicated that NAC could inhibit the autophagy induced by the CAP/DDP combination. All of these results demonstrated that the combination of CAP and DDP may trigger apoptosis and autophagy in OS cells by inducing ROS. Furthermore, our results also showed that ROS blockage by pretreatment with NAC could reverse the inactivation of AKT and mTOR in the AKT/mTOR pathway and inhibit JNK phosphorylation in the ROS/JNK signaling pathway. Taken together, all of the above results suggest that the combination of CAP and DDP triggers apoptosis and autophagy, which may be mediated by ROS through the inhibition of the ROS/Akt/mTOR pathway and activation of the ROS/JNK signaling pathway.

Finally, we further validated the effects of CAP and DDP in vivo by using xenograft models. Our results showed that tumor growth was effectively reduced in all treatment groups of 143B OS xenograft mice compared with that in the control group. In addition, the CAP/DDP combined treatment group showed the strongest inhibitory effects, consistent with the findings in vitro. Moreover, combined treatment with the two drugs did not significantly affect the body weight of the mice, and no significant nephrotoxicity were observed in any of the treatment groups of mice. These results indicated that this combination might be a relatively effective and safe regimen for OS.

## Conclusion

In summary, our present study demonstrated that CAP in combination with DDP was able to markedly inhibit OS cell viability and invasion and induce cell apoptosis, autophagy and G0/G1 cell cycle arrest in vitro and suppress xenograft tumor growth in vivo. In addition, the combination of CAP and DDP demonstrated a synergistic inhibitory effect on the viability of OS cells. The blockage of autophagic flux enhanced cell death, indicating that the autophagy induced by the combination treatment is a prosurvival process in cells. Our results also suggested that the underlying mechanisms of the effects of this combination treatment were initiated by excess ROS generation, which was followed by the activation of the ROS/JNK pathway and inhibition of the ROS/Akt/mTOR signaling pathway.

## Abbreviations

Baf-A1: Bafilomycin A1
CAP: Capsaicin
CI: Combination index
DDP: Cisplatin
MDC: Monodansylcadaverine
NAC: N-acetyl-L-cysteine
OS: Osteosarcoma
ROS: Reactive oxygen species
TEM: Transmission electron microscopy
Δψm: Mitochondrial membrane potential
